# Maiden Outbreak of Chikungunya in Dongguan City, Guangdong Province, China: Epidemiological Characteristics

**DOI:** 10.1371/journal.pone.0042830

**Published:** 2012-08-16

**Authors:** Zhang Qiaoli, He Jianfeng, Wu De, Wang Zijun, Zhong Xinguang, Zhong Haojie, Ding Fan, Liu Zhiquan, Wang Shiwen, Huang Zhenyu, Zhang Yonghui, Ke Changwen, Yuan Dakang, Liang Wenjia, Li Deqiong, Chen Pinghua

**Affiliations:** 1 Dongguan Municipal CDC, Dongguan, Guangdong, People's Republic of China; 2 Guangdong Provincial CDC, Guangzhou, Guangdong, People's Republic of China; 3 China CDC, Beijing, People's Republic of China; 4 Wanjiang Hospital, Dongguan, Guangdong, People's Republic of China; Blood Systems Research Institute, United States of America

## Abstract

**Background:**

This study was conducted to identify epidemiological characteristics of the first documented CHIK fever outbreak in China and evaluate the effect of the preventive measures taken.

**Methodology/Principal Findings:**

From September 1 to October 29, 2010, China's first documented outbreak of CHIK fever occurred in the Xincun community of Wanjiang District of Dongguan city, Guangdong province; 253 case-patients were recorded, of which 129 were laboratory confirmed, with an attack rate of 1%. Before September 18^th^ the number of CHIK fever cases remained relatively low in the Xincun community; from September 19^th^ onwards, the number of cases increased drastically, with an outbreak peak on October 4^th^. Cases were distributed across nine small village groups in the Xincun community, with an attack rate of 0–12% at the village level. The household attack rates ranged between 20% and 100%. No significant difference was found in the attack rate between males and females. There was a significant difference in the attack rate in different age groups (chi-square = 18.35, p = 0.005); highest in patients aged 60 years or older and the lowest in patients aged under 10. The major clinical characteristics of patients are fever (100%), joint pain (79%) and rash (54%). Phylogenetic analysis of the E1 gene on the five earliest confirmed cases showed that the strains of CHIKV isolated from their sera were highly homologous (up to 99%) with isogeneic strains isolated in Thailand in 2009. After control measures were taken, including killing adult mosquitoes and cleaning breeding habitats of *Aedes* mosquitoes, the Breteau index and Mosq-ovitrap index decreased rapidly, and the outbreak ended on October 29.

**Conclusion/Significance:**

The infection source of the outbreak was imported. Cases showed obvious temporal, spatial, and population aggregation during the outbreak. Comprehensive control measures based on reducing the density of *Aedes* mosquitoes were effective in controlling the epidemic.

## Introduction

CHIK fever is a viral disease mainly characterized by fever, rash, and severe joint pain. First isolated in Tanzania in 1952 [1], CHIKV is most commonly transmitted to humans through the bite of an infected *Aedes* mosquito. Recently, CHIK fever has become extensively prevalent in tropical and subtropical regions such as Asia, Africa, and Indian Ocean islands, and is an increasingly serious public health problem [2].

Dongguan City, between East longitude 113°31′ to 114°15′ and North latitude 22°39′ to 23°09′, is located in the central south of Guangdong province on the east coast of the Pearl River Estuary, with a modest subtropical monsoon climate and an annual average temperature of 23.6 degree Celsius. The land area of Dongguan is 2,465 square kilometers, and the permanent population is 8,220,237. It consists of four districts and 28 towns. In the past decade, the major mosquito-borne diseases in Dongguan City have been dengue fever and malaria. Cases of malaria are all sporadic, with *Anopheles sinensis* and *Anopheles anthropophagus* being the major transmission vectors. Dengue fever cases are most commonly sporadic imported cases, with *Aedes albopictus* being the only transmission vector.

**Figure 1:**
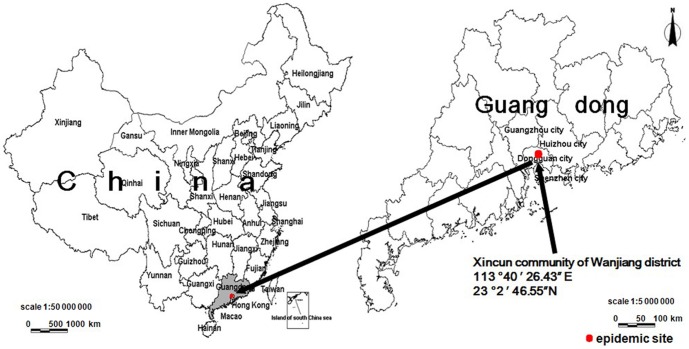
Location of the epidemic site of the CHIK fever outbreak at Xincun community.

The first imported sporadic case of the disease in China was reported in Xishuangbanna, Yunnan Province, in 1987, where the virus strain was isolated from the patient's blood [3]. Since then, all cases of CHIK fever in China have been imported, and no local case had been reported prior to the Dongguan outbreak [4]. The first documented community-based outbreak of CHIK fever in China was declared in the Xincun community of Wanjiang district in Dongguan city of Guangdong province (Figure 1) on October 2, 2010.

After the Xincun outbreak was confirmed, the Dongguan Municipal Center for Disease Control and Prevention (CDC), together with the Guangdong Provincial CDC and China CDC, conducted an epidemiological investigation and made suggestions for controlling the epidemic situation. The results of this investigation may be helpful in better understanding the epidemiological characteristics of this emerging infectious disease, and the control measures taken in the outbreak may be used as a reference for other regions dealing with CHIK fever epidemics in the future.

## Methods

### Study site

The Xincun community is a rural-urban fringe area surrounded by water on three sides. The area contains several still-water rivers, with 13 closely connected, small village groups under its jurisdiction. The total area of this rural-urban fringe is about 3.5 km^2^, with a population of about 11,000.

### Data sources

All cases were investigated by a purpose-designed epidemiological survey form. Data including demographic information, clinical data, contact history, and travel history of cases were collected.

### Cases identified and definitions

House to house visits by public health professionals and active reporting by clinical doctors were established to identify all possible cases during the outbreak. A suspected case was defined as a possible instance of CHIK fever in any person who met all of the following conditions: 1) living in Xincun community, Wanjiang District, Dongguan; 2) experienced sudden fever (body temperature >38 degrees Celsius) with at least one symptom of the disease, such as joint pain, rash or petechiae, and musculoskeletal pain since September 1, 2010; 3) did not undergo any confirmation test, or results of confirmation tests for both CHIKV nucleic acid and CHIKV IgM were found to be negative. A confirmed case was an instance of CHIK fever in any person 1) matching the above conditions, and 2) and whose blood tested positive for CHIKV nucleic acid and/or CHIKV IgM.

### Detection of CHIKV nucleic acid and CHIKV IgM

All investigations, sample collections, and tests in this study were conducted after obtaining informed consent from case-patients and approval from the Science and Ethics Committee of the Dongguan Municipal CDC.

A 5 mL sample of venous blood was collected from CHIK fever case-patients. Tests for CHIKV nucleic acid and for CHIKV IgM were simultaneously conducted on serum samples collected from patients within 7 days of onset of symptoms. CHIKV IgM tests were also made on serum samples collected from cases-patients discovered during the retrospective investigation. Sera were tested for CHIKV nucleic acid by real-time fluorescence quantitative RT-PCR kit (Shanghai ZJ Bio-Tech Co., Ltd., Shanghai, China (Batch no., 20101001-1; Expiry date, October 6, 2011; ABI 7500 fluorescence quantitative PCR). Analysis sensitivity was 1×10^3^ copies/mL, with CT≤30 considered as positive, 30<CT<35 as suspicious and CT≥35 as negative. The positive and negative controls of RT-PCR detection were provided by CHIKV RT-PCR kit. The negative controls were DEPC-H2O, and the positive controls contain high concentrations of target DNA (1×10^7^ copies/ml).CHIKV-specific antibodies were detected using an indirect immunofluorescence test (IIFT) (EUROIMMUN., Lübeck, Germany). In short, rheumatic factor was pre-adsorbed with EUROSORB reagent for the detection of IgM. The samples diluted 1∶10, and 25 µL were applied to the reaction fields of the BIOCHIPs, which were then incubated for 1 h. For antibody detection, anti-human IgM antibodies labeled with fluorescein isothiocyanate (FITC) were used. The positive and negative controls were provided by EUROIMMUN anti-CHIKV IIFT kit. The positive controls were human antibodies against CHIKV (IgM), and the negative controls were human antibodies negative against CHIKV. After the test finished, the samples were judged as negative if no reaction at 1∶10 diluted samples. The samples were positive if specific fluorescence pattern could be seen at 1∶10 diluted samples.

### Gene sequence analysis

The five earliest confirmed cases were selected for phylogenetic analysis of the *E1* gene. Extraction of viral RNA from the CHIKV isolates or human sera was done using a QIAamp Viral RNA Mini Kit (QIAGEN, Hilden, Germany) according to the manufacturer's instructions. The partial sequence of the gene encoding *E1* from CHIKV was amplified by reverse transcription-PCR using a QIAGEN OneStep RT-PCR Kit. Amplified PCR products of the *E1* region (325 basepairs) were purified and sequenced using an automated 3100 Genetic Analyzer (Applied Biosystems, Carlsbad, CA, USA) with the PCR primers, without further cloning. Subsequently, a BLAST search of the obtained sequence was run against GenBank to verify the amplified fragments. Alignment of the *E1* gene of CHIKV isolates and reference strains (Table 1) was performed using MEGA, version 4.0 [5]. The nucleotide sequence data reported in this study were deposited in the Genbank database with accession numbers HQ659773 and HQ392517-HQ392520.

**Table 1:** *E1* gene nucleotide sequences of the CHIKV strains used for phylogenetic analysis.

Strain	Genotype	Source	GenBank no.
IbH35-Nigeria-1964	West African genotype	GenBank	AF192893
37997-senegal-1983	West African genotype	GenBank	AY726732
0806a-TW-2008	Asian genotype	GenBank	FG807890
C03295-THA-1995	Asian genotype	GenBank	AF192897
IND-63-WB1-1963	Asian genotype	GenBank	EF027140
Ross-Tanzania -1953	CSE African genotype	GenBank	AF192905
18211-SA-1976	CSE African genotype	GenBank	AF192903
Ag41855-UGD-1982	CSE African genotype	GenBank	AF192907
ITA07-RA1-ITA-2007	CSE African genotype	GenBank	EU244823
HM369441-India-2009	CSE African genotype	GenBank	HM369441
EHIss622-Singapo-2008	CSE African genotype	GenBank	EU441883
chk10-Tha-2009	CSE African genotype	GenBank	FJ882911
GD112-HQ392517	CSE African genotype	This study	HQ392517
GD113-HQ392518	CSE African genotype	This study	HQ392518
GD114-HQ392519	CSE African genotype	This study	HQ392519
GD115-HQ392520	CSE African genotype	This study	HQ392520
GD124-HQ659773	CSE African genotype	This study	HQ659773

CSE: Central/South/East.

### Breteau index (BI) and Mosq-ovitrap index (MOI) surveillance

BI was used to measure the density of *Aedes mosquitoe* larvae and evaluate the effect of clearing breeding habitats of *Aedes* mosquitoes at the epidemic site. It was calculated as the number of containers positive for *Aedes* mosquito larvae per 100 houses. MOI was used to measure the density of adult *Aedes albopictus* and evaluate the effect of anti-adult treatments. This was calculated as the number of mosq-ovitraps positive for adult *Aedes albopictus* and *Aedes* mosquito larvae per 100 retrieved mosq-ovitraps.

### Preventive measures

A series of comprehensive control measures was applied in the Xincun community after the CHIK fever outbreak had been confirmed. These measures included spraying pesticide inside and outside of houses once per week to kill adult mosquitoes, door-to-door cleaning of *Aedes* mosquito habitats both indoors and outdoors, improving fever case surveillance with a CHIK fever case-reporting system, and public health education. Over 7,000 people in Dongguan City were mobilized to join the epidemic control of CHIK fever in the Xincun community.

### Statistical analysis

EpiData 3.02 (EpiData Association, Odense, Denmark) Microsoft Excel was used to process and analyze the data. Comparisons of ratios between different groups were conducted using the chi-square test, and a *p*-value<0.05 was considered significant.

## Results

### Intensity of the epidemic

During the outbreak, 253 case-patients were reported, of which 129 cases were laboratory confirmed and 124 were suspected cases, with an attack rate of 1% (129/11,000). In the 129 confirmed cases, 32 were confirmed by RT-PCR only (25% of total cases, 32/129), 52 by serologic analysis only (40%, 52/129), and 45 by both methods (35%, 45/129); 57 cases were found retrospectively (44%, 57/129). Among the 124 suspected cases, confirmation tests were not performed on 52 cases due to lack of a blood sample. The serum RT-PCR and serology test results of the remaining 72 cases were all found to be negative.

### Temporal distribution

**Figure 2:**
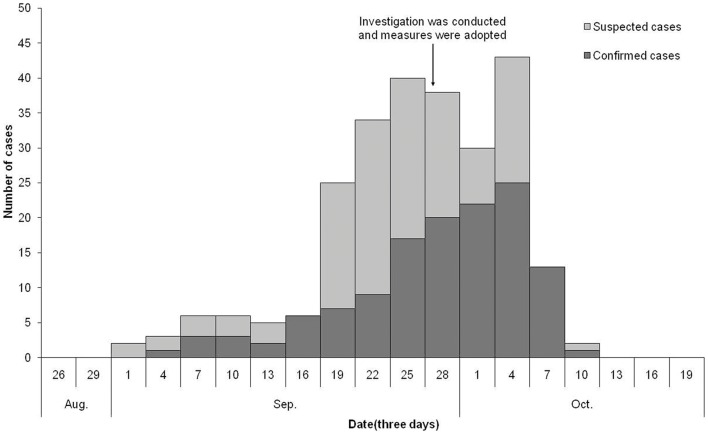
Temporal distribution of onset of cases during the CHIK fever outbreak in Xincun.

Retrospective investigation found that the earliest suspected case occurred on September 1, 2010. Before September 18^th^ the number of cases of CHIK fever remained relatively low; from September 19^th^ onwards, the number of cases increased drastically, with an outbreak peak on October 4^th^ (Figure 2). Four days after preventive and controlling measures were applied, the number of new cases decreased rapidly, and no new case occurred since the onset of the last case on October 10, 2010.

### Spatial distribution

Cases were distributed across nine small village groups in the Xincun community and attack rates at the village level were shown in Table 2. The earliest cases occurred in Jiangcun village group 1 and Jiangcun village group 2, which are at the eastern fringe of the community. The number of cases in these two village groups was high, accounting for 33% (42/129) and 26% (34/129) of the total, respectively. The attack rates in these two groups were significantly higher than those in the other seven small village groups (chi-square = 475.81, p = 0.000).

**Table 2:** Attack rates of CHIK fever confirmed cases in different villages in the Xincun community.

Village	Jiangcun 1	Jiangcun 2	Xiajiao 1	Cuntou	Luwu	Xiajiao 2	Laiwu	Xinningji	Cunwei
No. Cases	42	34	12	12	11	6	7	4	1
Population	373	287	939	1944	1090	943	1327	410	1054
Attack Rate (%)	11	12	1	1	1	1	1	1	0

Cases were distributed across 87 houses, 28 of which had 2 or more cases, accounting for 32% (28/87) of the number of houses with cases. The household attack rate ranged between 20% and 100%.

### Population distribution

Of the 129 confirmed cases, 59 were male, with an attack rate of 1% (59/5612), and 70 were female, with an attack rate of 1% (70/5388). No significant difference was found in the attack rate between males and females (chi-square = 1.46, p = 0.227). Patients were aged 3–93 years (mean age, 42 years). There was a significant difference in the attack rate among different age groups (chi-square = 18.35, p = 0.005) (Table 3).

**Table 3:** Age distribution for confirmed cases during the CHIK fever outbreak in the Xincun community.

Age (years)	No. cases	Population	Attack rate (%)	Percent of total
<10	5	810	1	4
10 to <20	17	1818	1	13
20 to <30	15	2048	1	12
30 to <40	22	1584	1	17
40 to <50	23	1843	1	18
50 to <60	15	1365	1	12
60	32	1532	2	25
**Total**	129	11000	1	100

Most cases (54%, 70/129) were in people whose main occupation was housework or who were unemployed, followed by workers (18%, 23/129) and students (16%, 20/129).

### Clinical Characteristics

The major clinical characteristics of patients with CHIK fever are fever, joint pain and rash (Table 4). Routine hematological testing was conducted on the 33 confirmed cases, with 8 patients exhibiting leukopenia (24% of total cases, 8/33) and 1 patient with thrombocytopenia (3%, 1/33). All cases recovered, with no deaths.

**Table 4:** Clinical manifestations of CHIK fever confirmed cases in the Xincun community.

Sympton	No. Cases	Occurrence(%)
Fever	129	100
Joint Pain	102	79
Rash	70	54
Muscle Pain	59	46
Headache	40	31
Face Flushing	29	22

### Source of infection

**Figure 3:**
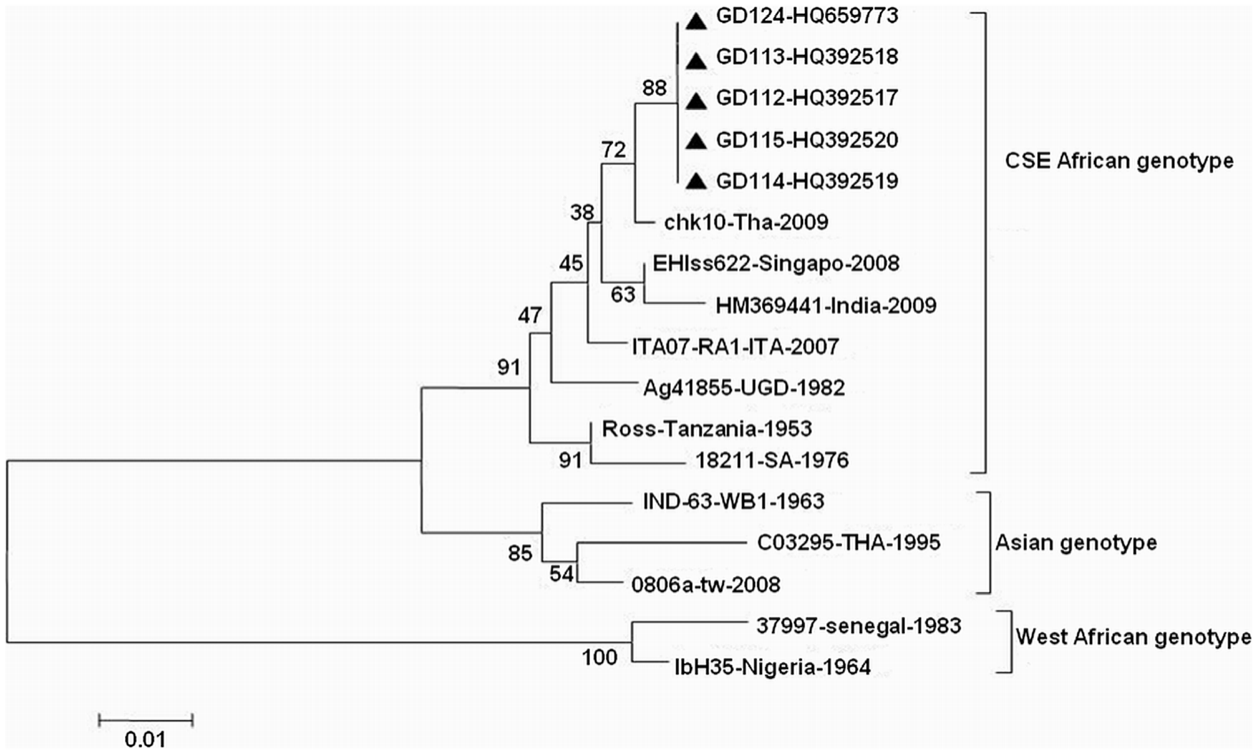
Phylogenetic dendrogram of 5 Dongguan CHIKV strains. This dendrogram was constructed using the neighbor-joining method based on the alignment of *E1* gene sequences. The bootstrap values from 1000 pseudoreplicates for major lineages within the tree are shown as percentages. Dongguan CHIKV strains are marked using the symbol ▴.

There are three known genotypes of CHIKV according to the divergence of the *E1* gene sequence: the West African, Asian, and Central/South/East (CSE) African genotypes [6], [7]. Dongguan CHIKV strains isolated from patients in this outbreak belonged to the CSE genotype, which has been prevalent in Africa in the past 50 years and has now spread to many countries on other continents (Figure 3).

In this study, none of the patients or their family members had traveled to Southeast Asia or Africa, or had contact with any person with a suspected case of CHIK fever outside the community in the month before the onset of the disease.

The results of gene sequence analysis and alignment to partial serum samples showed that the CHIKV strain in the Dongguan outbreak was similar to the epidemic strain of CSE, and that the highest homology at the nucleotide level between strains in this study and the isogeneic strain chk10-Tha-2009 (GenBank number FJ882911) isolated in Thailand in 2009 was 99%.

### BI and MOI surveillance

**Figure 4:**
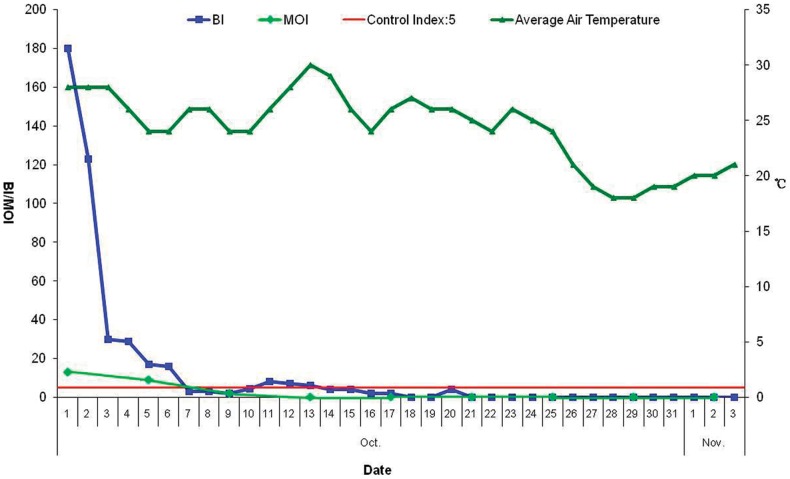
Daily changes in BI, MOI and air temperatures during the CHIK fever outbreak in Xincun.

The BI and MOI for the Xincun community were 180 (90/50×100) and 13 (7/53×100), respectively, before control measures were applied. However, both BI and MOI decreased rapidly after control measures were taken from October 2. BI remained below 5 from October 14 and MOI remained below 5 from October 9 (Figure 4).

### Evaluation of the effect of preventive and control measures

No new cases occurred during the 19 days from the onset of the last case on October 10 to October 29, and the BI and MOI remained <5 for over two weeks, marking the end of the outbreak.

## Discussion

No cases of CHIK fever had been reported in Dongguan before the Xincun outbreak, and only sporadic imported cases have been reported in Yunnan Province and the city of Guangzhou [3], [8]. Delayed reporting was observed in the Xincun outbreak. The number of cases of CHIK fever increased greatly from September 19, 2010; however, it was not until September 30 that the Dongguan Municipal CDC received reports of the outbreak. The center started an investigation on October 1, and the outbreak was confirmed at 1:00 AM on October 2. The delay in reporting may have led to the subsequent spread of cases throughout the Xincun community and made the task of controlling the outbreak more difficult. This suggests that it is necessary to improve the training of primary care medical staff to increase their ability to detect and report emerging infectious diseases.

In India during the CHIK fever epidemic of December 2005 to April 2006 the attack rate was reported as high as 45% in some regions [9] and during the epidemic on Reunion Island of France, 2005–2006, the overall attack rate was 35% [10]. In the Xincun outbreak, the attack rate was not as high as those two epidemics, which may be attributed to the relatively earlier discovery of the epidemic as well as the effective enforcement of control measures.

Where the epidemic occurred for the first time in other countries, no difference was found in the attack rate between the sexes and among different age groups [11]. However, in regions with reoccurrence of the CHIK epidemic, females and adults were usually found to have a higher attack rate [10], [12]. In the Xincun outbreak, again, no difference was found in the attack rate between the sexes. People who spent much of their time at home were probably more at risk of mosquito-borne disease, particularly during periods of mosquito activity during the day, which may explain the differences in attack rates by age and occupation in the Xincun outbreak.

None of the patients or their family members had traveled to Southeast Asia or Africa. The Xincun outbreak was considered a local outbreak of CHIK fever caused by an imported case, although the source is unclear. This conclusion is supported by the following. First, no local epidemic of CHIK fever had previously been reported in China. Second, there were articles that reported that some CHIK fever patients appeared to be asymptomatically infected but could transmit the disease [13], [14]. Third, many people travel between Dongguan, Africa and Southeast Asia. According to data from the Exit and Entry Administration Department, 13,000 people entered Dongguan from Southeast Asia and Africa in 2009, and almost 60,000 Dongguan residents visited these regions. Fourth, gene sequence analysis and genetic relatedness of strains from Xincun with Thai CHIKV of confirmed cases also supports that the outbreak was caused by an imported case or vector.

Dongguan is an area of frequent external contacts, with high population density and material flows, and it is located in the sub-tropical zone. These are the main reasons contributing to the outbreak of CHIK fever. The surveillance on vector conducted by Dongguan Municipal CDC in the past ten years showed that *Aedes albopictus* has being the only *Aedes* in Dongguan city. So, it was concluded that *Aedes albopictus* was a probable vector causing the outbreak. After control measures were taken, the density of *Aedes albopictus* decreased quickly, while temperatures in the region maintained at 24–30 degree Celsius suggesting that control measures, and not a temperature drop, were responsible for effectively decreasing the density of *Aedes albopictus* and controlling the epidemic.

No criteria for defining the end of a CHIK fever epidemic outbreak could be found in the literature. In the report of 2 cases of locally transmitted CHIK fever that occurred in southeastern France, Grandadam M uses the observation of no occurrence of any new case 45 days after the onset of the last case as the criterion for determining epidemic termination [15]. In this study, the following criteria were used to determine the endpoint of this particular epidemic outbreak: no new cases 19 days since the onset of the last case and the local BI and MOI remaining below 5 for over 2 consecutive weeks [16].

Vertical transmission of CHIKV in infected *Aedes* mosquitoes could lead to rapidly increased numbers of mosquito vectors carrying the virus and is the main reason for the rapid spread of the epidemic. In addition, mosquito eggs are very hardy in the external environment and can even survive through the winter [17]. This might be a reason why CHIKV becomes endemic to many regions after the first outbreak. It will be worth observing whether CHIK fever becomes endemic to Dongguan.

It was reported that after 7–8 years of absence, re-emergence of a CHIK outbreak could occur unpredictably: for example, Africa in 1999 and 2000 in the Congo [18] and Asia in 2001 in Java [19]. Therefore, it is very important to reinforce surveillance of CHIK in order to detect any epidemic early and take timely control measures.
